# On the Application of Artificial Teeth, on Plates with Clasps

**Published:** 1845-12

**Authors:** S. M. Shepherd


### ARTICLE V.

### *On the Application of Artificial Teeth, on Plates with Clasps.*

By S. M. SHEPHERD, D. D. S.

MESSRS. EDITORS :—As improvement has been the all-absorbing theme in the dental profession for the last several years, and especially so since the establishment of a Dental Journal, through which the various members can report their experiments, discoveries, &c., each item, I may say, in the catalogue of dental operations, medical, surgical and mechanical, has been the subject of close *re-examination* and experiment. But while all who are at all familiar with the subject are satisfied that numerous and valuable discoveries and improvements have been made, none ought to doubt that in some particulars, errors have crept in. This remark is applicable to errors in practice, wherever found; but my object at this time is to call the attention of the profession to one single item, the manner of clasping artificial teeth. I think a decided error prevails in this particular, which has done, and is still doing a great deal of damage. The error of which I speak is found in the broad clasp, which has become almost universal.

Since the appearance of Dr. Brown's most valuable essays on "mechanical dentistry," accompanied by his plates, I observe that almost every artificial piece requiring clasps is copied after his ; an error the more fatal because so few were to be found in the whole of his publications on the subject. And it is a singular fact, that, not an idea suggested by him has been, so far as I am able to judge, more readily seized upon. Those papers of the doctor's were well timed, and served as a sort of pioneer in the mechanical branch of dentistry, especially among dentists who are situated remote from the large cities, who seldom see an artificial piece except of their own manufacture. Many labored under great embarrassment, for want of knowledge, which is amply furnished in those papers.

But no practice is admissible which results in injury ; and so far as my observation extends, the broad clasp, with few exceptions, does inflict injury on the tooth over which it passes.

A single reflection upon the articulation of the teeth will show the impropriety of placing any clasp or fixture on them that would prevent their natural motion. The periosteum or the membranes lining the socket and covering the fangs of the tooth, gives it the power of motion, of which, it may not on any account be robbed.

This is done to a greater or less extent by the use of the broad clasp.

A description of the manner in which the clasp is made, of which I complain, will give some idea of its unyielding character. The plate is cut and stamped down upon the model, extending not merely to the tooth, but having a circular notch cut or filed out so as to conform exactly to the shape of the tooth at its neck, one-third, or even half way round it. The clasp is then cut from a piece of plate a little thicker than ordinary, and being wrapped around the tooth, covering nearly its whole crown, the edge is neatly fitted down to the plate and soldered firmly. This constitutes a fixture as unyielding, comparatively, as the nose band of a cart axle, and when forced over the crown of the tooth, it holds it with something like the firmness of a vice. But in many instances, when these cart bands come to be applied to the teeth, they do not fit; they were made to fit the model precisely, and unless the model is an exact representation of the teeth, they do not fit the teeth of course. But fit or not, if they will go on at all, and *stay* there, they are usually forced on; for the manner of their construction will not admit of much alteration after the piece is finished, no matter how much or how little they may require it. In case the fit is not perfect, the position of the tooth may be changed, pushed over a little to one side, which gives it a very uneasy feeling until the periosteum becomes paralysed at the points of pressure. Thus the circulation in this delicate membrane is cut off at those points, and disorganization is the inevitable consequence.

Another important consideration arises here; the fit of these clasps is usually so *tight* that the wearer finds it exceedingly difficult to remove them for the purpose of cleaning, so much so that in many cases it is partially, and in some, wholly neglected. Wherever this is the case, sad is the result.

I will mention one case only, out of many which have fallen under my notice. Mrs. ———, of the town in which I live, visited one of our northern cities, a few years since, for the purpose of having some artificial teeth inserted. She had lost the superior central and lateral incisors, the cuspidati, and the first and second bicuspids of both sides. I saw the mouth before she went on, and it was a most beautiful case to be filled. The teeth to be clasped to, were well adapted to the purpose, perfectly healthy and firmly set in their places. She went on, however, and had them put in. Some twelve or fifteen months after this she called on me to examine her mouth, and said her teeth required some little alteration. I saw in an instant that very broad and powerful clasps had been used, and all round these, originally healthy molar teeth, the gums were in a state of actual suppuration, and highly inflamed. I removed the artificial teeth, but not without considerable difficulty, so very tightly were they forced on; and when I did succeed, the blood flowed freely from the gums. I asked the lady how *she* managed to get them out when she wanted to clean them.

"I get them out!" said she; "they have never been out but once since they were in, and then I got a dentist to take them out to do something to them;" and no one would doubt it after looking in her mouth. Those fine, healthy molar teeth, which I saw not more than two years before, were now partly drawn from their sockets, quite loose, and bid fair to make a final exit in less than two years more.

The idea seems to be prevalent, that the more firmly an artificial piece can be secured in the mouth, the better.

It is certainly an unpleasant thing to have one's teeth falling about in the mouth, of their own accord, and to see, when in conversation with an individual, his or her teeth drop down a quarter of an inch every time the mouth opens, it requires more patience than I possess to sit or stand still. So, then, for the comfort of all parties, it is necessary, when artificial teeth must be worn, to have them at least sufficiently secured to maintain their position.

This I think may be done by the use of an elastic clasp, which will not produce the injury to which I have referred. The clasp which I use is a wire, made nearly flat on one side, but a little more convex on the other. One end of this is soldered to the plate at a point where it will lie easy, and the other end extends to the tooth to which it is to be fastened, passes quite round, and is bent in, so as to spring to the tooth binding upon its surface, at least three-fourths of its circumference; and at the same time I give it a shape that will cause it to spring the plate to the roof of the mouth. Thus I have a clasp that acts as a spring, which is perfectly elastic and yet it is steady. I have used the various clasps which I have seen in use, and these in all respects please me better than any I have ever tried. When made right, I believe, they are less liable to become loose than any other, and if from any cause they should become loose, it is no trouble to make them fast. Some object to clasps of this kind that they are liable to wear the teeth off, around which they pass. Dr. Westcott ridicules the idea of wearing off a tooth with a gold wire. There is a good deal of truth in his remarks on that subject, but I am not prepared to go all the way with him; for I am satisfied that I have seen some teeth cut off by the clasp alone; and that by a wearing process. So far as the clasp serves as a means of confining corrosive agents to the surface of the teeth, the narrow clasp is far less objectionable than the other.

The clasp that is most liable to wear away the tooth is that which does not pass sufficiently far around it to embrace it within itself; but simply passes behind it, while some other part of the piece rests against the front part of some other tooth, which thus for a time will keep it tolerably secure; but so soon as either of the teeth yield a little, which they will both do in a very short time usually, the whole piece becomes loose and begins to slip about in the mouth at every motion of the tongue. Then begins the wearing process; and if you tighten it by bending in the clasp, the teeth yield still further, and thus it continues until the tooth is cut off.

I will venture to affirm that this will never happen where the clasp embraces the tooth sufficiently to sustain itself independently of any other tooth.*

* Our own observations and experience do not accord with the views of Dr. S. as expressed in the foregoing article. Wide clasps when accurately adapted to the teeth on which they are placed, we have found, hold a dental substitute more firmly than narrow ones, and that too, with less injury to the teeth, and it is for this reason, that the former have almost wholly, within the last few years, taken the place of the latter; we are glad, however, that the attention of the profession is invited to the subject, and should be glad to have a full expression of opinion from the members of the profession upon it.—*Balt. Ed.*

